# Different Frequencies of Drug Resistance Mutations among HIV-1 Subtypes Circulating in China: A Comprehensive Study

**DOI:** 10.1371/journal.pone.0091803

**Published:** 2014-03-24

**Authors:** Hongshuai Sui, Tao Gui, Lei Jia, Wei Guo, Jingwan Han, Yongjian Liu, Zuoyi Bao, Hanping Li, Jingyun Li, Lin Li

**Affiliations:** 1 Department of AIDS Research, State Key Laboratory of Pathogen and Biosecurity, Beijing Institute of Microbiology and Epidemiology, Beijing, China; 2 Clinical Laboratory of Beidaihe 281 hospital, Qinhuangdao, Hebei province, China; Shanghai Medical College, Fudan University, China

## Abstract

The rapid spreading of HIV drug resistance is threatening the overall success of free HAART in China. Much work has been done on drug-resistant mutations, however, most of which were based on subtype B. Due to different genetic background, subtypes difference would have an effect on the development of drug-resistant mutations, which has already been proved by more and more studies. In China, the main epidemic subtypes are CRF07_BC, CRF08_BC, Thai B and CRF01_AE. The depiction of drug resistance mutations in those subtypes will be helpful for the selection of regimens for Chinese. In this study, the distributions difference of amino acids at sites related to HIV drug resistance were compared among subtype B, CRF01_AE, CRF07_BC and CRF08_BC strains prevalent in China. The amino acid composition of sequences belonging to different subtypes, which were obtained from untreated and treated individuals separately, were also compared. The amino acids proportions of 19 sites in RT among subtype B, CRF01_AE and CRF08_BC have significant difference in drug resistance groups (chi-square test, *p*<0.05). Genetic barriers analysis revealed that sites 69, 138, 181, 215 and 238 were significantly different among subtypes (Kruskal Wallis test, *p*<0.05). All subtypes shared three highest prevalent drug resistance sites 103, 181 and 184 in common. Many drug resistant sites in protease show surprising high proportions in almost all subtypes in drug-naïve patients. This is the first comprehensive study in China on different development of drug resistance among different subtypes. The detailed data will lay a foundation for HIV treatment regimens design and improve HIV therapy in China.

## Introduction

30 years has passed since human immunodeficiency virus (HIV) was confirmed as the pathogen of acquired immunodeficiency syndromes (AIDS) [1].This pandemic have already spread all over the world, infected millions of people and caused enormous social and economic loss. Highly active antiretroviral therapy (HAART) offensively suppressed HIV replication *in vivo*, prolonged life of HIV infected persons and promote their live quality, however, the occurrence of drug resistance impair this effect greatly and has become the main cause of antiretroviral therapy (ART) failure [2].

High diversity was the main characterization of HIV. Two types of HIV, HIV-1 and HIV-2, were identified based on the genetic difference. The former is the main type that has caused global pandemic and representing absolutely most of the HIV isolates, while the latter is restricted in western and central Africa and very few cases out the continent only account for no more than 2% of all HIV infection [3]. HIV-1 contains four groups (M, N, O and P) [4]–[6], group M is the major group responsible for the AIDS pandemic which including nine pure subtypes (A to D, F to H, J and K) as well as more than 50 circulating recombinant forms (CRFs) and many unique recombinant forms (URFs) [7]. So far, all antiretroviral drugs were designed based on subtype B for it is the major subtype prevalent in the US and Western Europe, but this subtype caused only approx. 10% of all HIV infection [8]. High level of genetic polymorphisms was found among HIV sequences belonging to different subtypes, which may cause the frequencies of drug resistance mutations different among different subtypes. Illustration of the difference will be helpful to analyze why and how drug resistance mutations appear and meaningful for clinic individualized antiretroviral therapy.

Among the 9 antiretroviral drugs freely provided by the Chinese government presently, zidovudine(AZT), lamivudine(3TC), stavudine(d4T), didanosine(ddI) and tenofovir(TDF) belong to nucleoside reverse transcriptase inhibitors (NRTIs); efavirenz(EFV) and nevirapine(NVP) belong to non-nucleoside reverse transcriptase inhibitors (NNRTIs); indinavir/r(IDV/r) and lopinavir/r(LPV/r) belong to protease inhibitors (PIs) [9]. The first line ART regimens were usually composed of two NRTIs (usually contain two of d4T, AZT or 3TC) and one NNRTIs of NVP or EFV. The other drugs always act as substitutes used for those who failed ART or some special populations as pregnant women and those who co-infected with other diseases as tuberculosis, HBV and so on [9], [10]. Generally, viral subtypes at a population level were always not considered during ART regimens selection, despite the genetic difference among HIV-1 subtypes may confer differently to the development of drug-resistance.

In this study, HIV-1 strains belonging to main subtypes prevalent in China, including subtype B, CRF01_AE, CRF07_BC and CRF08_BC, were collected to investigate the difference of drug resistant mutations among different subtypes. Furthermore, the correlations between specific drug-resistant mutations and subtypes were explored, which may be helpful in alerting and adjusting therapeutic schedule to promote the effect of antiretroviral treatment.

## Materials and Methods

### HIV-1 sequences

The pol region, especially the region coding protease and the start 720 base pairs of reverse transcriptase (RT), is always used for HIV genotype drug-resistance test. Three kinds HIV-1 sequences of that region were included in this study: i) interested sequences fragments downloaded from the Los Alamos HIV sequences database (http://hiv-web.lanl.gov). All problematic sequences were excluded and only one sequence was selected per patient; ii) sequences generated in our laboratory for annual drug resistance surveillances and epidemiological analysis in the past 3 years were also included. All sequences were produced by nest RT-PCR with plasma viral RNA as template, then determined with pyrosequencing method; iii) sequences provided by the AIDS Care Center of the Yunnan Infectious Disease Hospital during drug resistance surveillance in 2012. To ensure data quality, sequences that contain stop codons and individual resistance codons with more than 2 ambiguities base per nucleotide position or with 2 ambiguities bases, which may be the result of contamination in RT-PCR or bad sequencing, were excluded from the analysis.

### Sequences subtyping and drug resistance evaluation

Subtypes of the sequences from the HIV sequence database were already determined in the database and downloaded directly. Sequences generated in our laboratory and those provided by the AIDS Care Center of the Yunnan Infectious Disease Hospital were subtyped online with REGA HIV-1 subtyping tool 3.0(http://www.bioafrica.net), which incorporates both phylogenetic and boot scanning methods in an automated process to identify subtypes, CRFs and URFs of query sequences [11], [12]. MEGA 5.1 was used to perform phylogenetic analysis to identify the subtypes of sequences which could not be determined with REGA. All sequences of subtype B, CRF01_AE, CRF07_BC and CRF08_BC were included into drug resistance evaluation, which were performed online at HIV drug resistance database (http://www.hivdb.stanford.edu).

Sequences were sorted into several groups based on subtypes and drug resistance, i) resistant sequences group was make up of all four subtypes sequences with any major and/or minor drug resistant mutation(s); ii) susceptible sequences groups were composed of sequences containing no drug resistant mutations, and sequences were further subdivided into three small groups according to ART conditions: a) no background information group, which contains the sensitive sequences download from the HIV sequence database with little background information and no clear data about ART, those sequences were excluded from further analysis; b) drug naïve susceptible group, which contains sensitive sequences isolated from patients never expose to ART drugs; c) ART susceptible group, which contains sensitive sequences isolated from patients who received ART.

### Drug resistant mutations

Drug resistant mutations were defined as any differences from the consensus B amino acid sequence [8] and selected mainly based on HIV drug resistance database and published data including all listed major mutations, minor mutations and other mutations which are polymorphism sites or occurs in combination with mutations obviously influence drug resistance level. A polymorphism is defined as a mutation that occurs in at least 1% of a population not exposed to selective drug pressure [13], [14]. Drug resistance mutation sites are selected based on drugs used in China as follows: NRTIs resistance related sites 40, **41**, 44, 62, **65**, 66, **67**, 68, **69**, **70**, 71, **74**, 75, 77, **115**, 116, 118, **151**, **184**, **210**, **215** and **219**; NNRTIs resistance related sites 98, **100**, **101**, **103**, **106**, 108, **138**, 179, **181**, **188**, **190**, 225, 227, **230**, 236 and 238; PIs resistance related sites 10, 11, 20, 23, 24, **30**, **32**, 33, 35, 36, 43, **46**, **47**, **48**, **50**, 53, **54**, 58, 63, 71, 73, 74, **76**, 77, **82**, 83, **84**, 85, **88**, 89, **90** and 93. Bolded numbers are major drug resistance mutation sites which can lead to drug resistance without any other mutations, while minor drug resistance mutation sites cannot cause drug resistance alone but always occur with major drug resistance mutations to decrease virus susceptibility and/or increase drug resistance level [15].

### Statistical analysis

Sequences were aligned with BioEdit v7.0 and manually edited before translating into amino acids. The frequencies of amino acids at every drug resistance site were calculated. To analyze the differently distribution of amino acids among different subtypes, chi-square or fisher's exact test were fulfilled with SPSS v17.0 software. If positive results were observed, Kruskal Wallis test was performed to ascertain whether the difference was due to genetic barriers of codons.

## Results

### HIV-1 sequences overview

A total of 3624 sequences were downloaded from HIV sequences database, 1483 sequences were generated in our laboratory and 335 sequences were provided by the AIDS Care Center of the Yunnan Infectious Disease Hospital, and there were 1984 subtype B, 1696 CRF01_AE, 502 CRF07_BC and 924 CRF08_BC sequences respectively included in this study after exclude those cannot meet the demand. For analytic convenience, nucleotide(s) insertion and deletion comparing to subtype B consensus sequence were omitted, the whole sequence was separated into protease region (amino acid: 1–99) and RT region (amino acid: 1–240) (Table 1).

**Table 1:** Sequences summary.

Subtype	resistance		no background sensitive		drug naïve sensitive		ART sensitive	
	Protease	RT	Protease	RT	Protease	RT	Protease	RT
subtype B	32	700	1422	1207	104	99	175	398
CRF01_AE	30	152	1659	1725	426	421	549	550
CRF07_BC	4	27	441	428	57	57	145	126
CRF08_BC	7	120	940	804	206	202	590	484

### Different distribution of drug-resistant mutations is observed among subtype B, CRF01_AE and CRF08_BC

**Figure 1:**
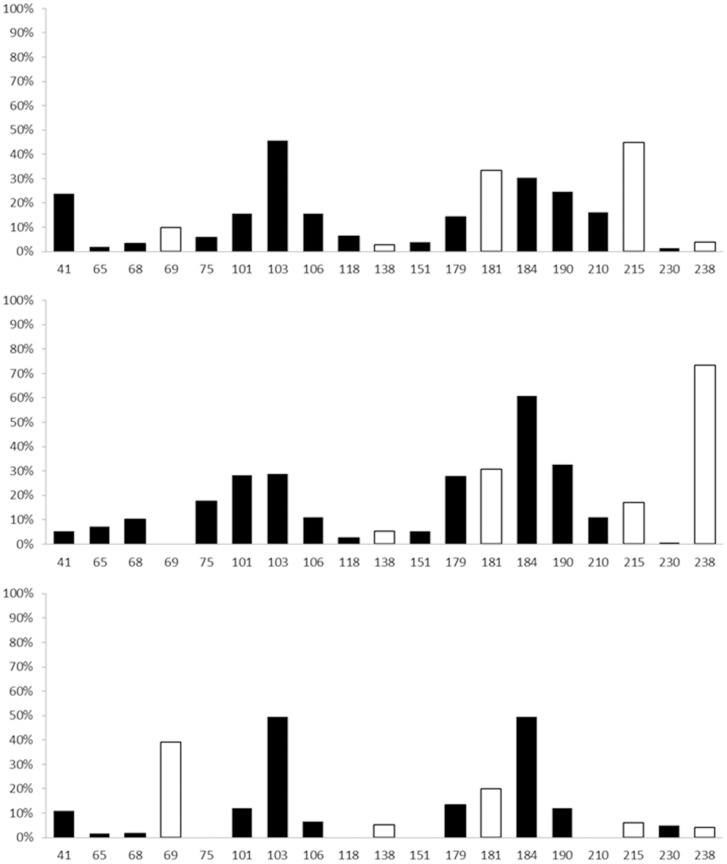
Subtype-specific drug resistance mutation sites analysis. Note: horizontal numbers indicate drug resistance mutation sites in RT region with significant difference among subtypes, vertical percentage indicate the proportion of mutated amino acids differ from the subtype B consensus sequence. Black columns means only amino acids distribution with significant difference among subtypes (chi-square or fisher's exact test, *p*<0.05), white columns means both mutated amino acids distribution and genetic barriers with significant difference among subtypes (Kruskal Wallis test, p<0.05).

To analyze the distribution of drug-resistant mutations among different subtypes, 38 sites locating in RT region were analyzed (PIs-resistant mutations were not analyzed because only 73 PIs-resistant sequences were obtained totally, especially CRF07_BC and CRF08_BC with only 4 and 7 sequences respectively). All drug-resistant mutations identified based on subtype B were found in at least one of the other two subtypes. Significantly different distributions were observed in 19 sites among subtype B, CRF01_AE and CRF08_BC (CRF07_BC was not considered due to a small sample size, chi-square or fisher's exact test, *p*<0.05). Among of them, three sites, including 103, 181 and 184, were found mostly prevalent (more than 20%) in all of three subtypes. The distributions of other drug resistance mutations were differently among subtypes, sites 41, 69, 101, 106, 179, 190, 210 and 215 were dominant in subtype B, while sites 68, 69, 75, 101, 106, 179, 190, 210, 215 and 238 in CRF01_AE and sites 41, 69, 101, 179 and 190 in CRF08_BC, which showed higher prevalent ratios than 10% respectively (Figure 1). Further analysis on codons of those sites show that synonymous mutation of amino acids caused by single base mutation were exist in all sites except sites 41,184 and 230, while nonsynonymous mutation of amino acids from 1 to 4 in different sites caused by different number of bases mutation were existed in almost all sites. Some usually occurred mutations like M41I, K65N, V75AS, K101P, E138K, Q151L, V179F and G190EQ were not observed in this study. Genetic barriers, which were defined as the number of bases change for a virus to develop resistance and escape select pressure from special drugs [16], were believed to play certain roles in the development of HIV drug resistance. In this study, 5 sites (69, 138, 181, 215 and 238) among the 19 sites being analyzed were shown to contain significant differences among subtypes (Kruskal Wallis test, *p*<0.05) (Table 2). This observation may explain why drug resistance develops at a similar rate during the antiretroviral therapy in China irrespective of subtype prevalence. In addition, only few of minor drug resistance mutation sites as 179 in subtype B, 75, 179, 238 in CRF01_AE and 179 in CRF08_BC account for preponderant proportion in corresponding subtypes compared that almost all major drug resistance mutation sites play dominant roles in all subtypes.

**Table 2:** Prevalence of wild type codons and genetic barriers of drug resistance mutation sites.

position	substitution	wt codon	WT codon proportion (%)			rt codon	required mutation	*p* ^1^	*p* ^2^
			B(700)	01_AE(152)	08_BC(120)				
41	M41L	ATG	74.1	94.7	87.5	CTG/TTG	1 tv	<0.001	0.136
65	K65R	AAA	94.2	90.8	2.5	AGA	1 ts	0.006	1.000
		AAG	3.3	1.3	95.0	AGG	1 ts		
68	S68G	AGT	94.3	9.2	90.8	GGT	1 ts	0.014	1.000
	S68N	AGC	1.6	77.0	3.3	AAT	1 ts		
69	T69N	ACT	87.9	11.2	58.3	AAT/AAC	1 tv	<0.001	<0.001
	T69S	ACC	-----	73.0	----	AGT	1 tv		
	T69D					GAT	1 ts, 1 tv		
75	V75I	GTA	92.9	69.1	97.5	ATA	1 ts	<0.001	0.498
	V75M	GTG	----	10.5	0.8	ATG	1 ts		
	V75T					ACA	2 ts		
101	K101E	AAA	81.3	64.5	80.8	GAA	1 ts	0.009	0.116
	K101Q	AAG	1.7	3.9	3.3	CAA	1 tv		
	K101R					AGA	1 ts		
103	K103N	AAA	50.4	66.4	38.3	AAC/AAT	1 tv	<0.001	0.627
	K103R	AAG	1.4	2.0	----	AGA	1 ts		
	K103S					AGC	1 ts, 1 tv		
106	V106I	GTA	80.5	70.4	90.0	ATA	1 ts	0.013	0.088
	V106A	GTG	1.6	12.5	1.7	GCA	1 ts		
	V106M					ATG	1 ts		
118	V118I	GTT	27.0	82.9	97.5	ATT	1 ts	0.079	0.464
		GTC	63.2	9.2	0.8	ATC	1 ts		
		GTA	1.4	1.3	0.8	ATA	1 ts		
138	E138Q	GAG	93.8	91.4	87.5	CAG	1 tv	0.028	<0.001
		GAA	2.9	2.0	4.2	CCG	2 tv		
151	Q151M	CAG	92.5	86.8	94.2	ATG	2 tv	0.025	----
		CAA	2.4	5.9	2.5	ATG	2tv, 1 ts		
179	V179I	GTT	76.7	63.2	77.5	ATT	1 ts	0.002	0.840
	V179D	GTG	3.3	2.0	----	GAT	1 tv		
	V179E	GTC	2.9	4.6	3.3	GAA/GAG	2 tv/1 tv		
	V179T					ACT	2 ts		
181	Y181C	TAT	64.6	65.1	76.7	TGT/TGC	1 ts	0.029	0.012
	Y181V	TAC	1.1	2.0	----	GTT	1 ts, 1 tv		
	Y181I					ATT	2 tv		
184	M184V	ATG	68.7	37.5	50	GTG/GTA	1 ts/2 ts	<0.001	0.823
	M184I					ATA	1 ts		
190	G190A	GGA	71.6	57.2	75.0	GCA	1 tv	0.058	0.101
	G190S	GGC	1.9	3.3	6.7	AGC	1 ts		
		GGG	----	----	2.5	GCA/AGC	1 tv/1 ts		
210	L210W	TTG	74.8	77.6	1.7	TGG	1 tv	<0.001	1.000
	L210S	TTA	6.5	7.2	96.7	TCG	1 ts		
		CTA	----		0.8	TGG/TCG	1 tv/1 ts		
215	T215Y	ACC	52.8	----	88.3	TAC/TAT	2 tv	<0.001	<0.001
	T215F	ACT	----	78.3	----	TTC/TTT	1 ts, 1 tv		
230	M230L	ATG	98.6	98.7	94.2	CTG/TTG	1 tv	0.038	1.000
238	K238T	AAA	94.0	25.0	89.2	ACA	1 tv	<0.001	<0.001
	K238N	AAG	0.9	0.7	4.2	AAC	1 tv		
	K238R					AGA	1 ts		

Abbreviations: wt – wild type; rt – resistance type; ts – transition; tv – transversion; underlined bases are those mutated and always lead to drug resistance; *p* ^1^ was calculated use chi-square or fisher's exact test; *p* ^2^ was calculated with Kruskal Wallis test.

### Polymorphism in sites related to drug-resistance was found differently among subtypes in drug-naïve individuals

**Figure 2:**
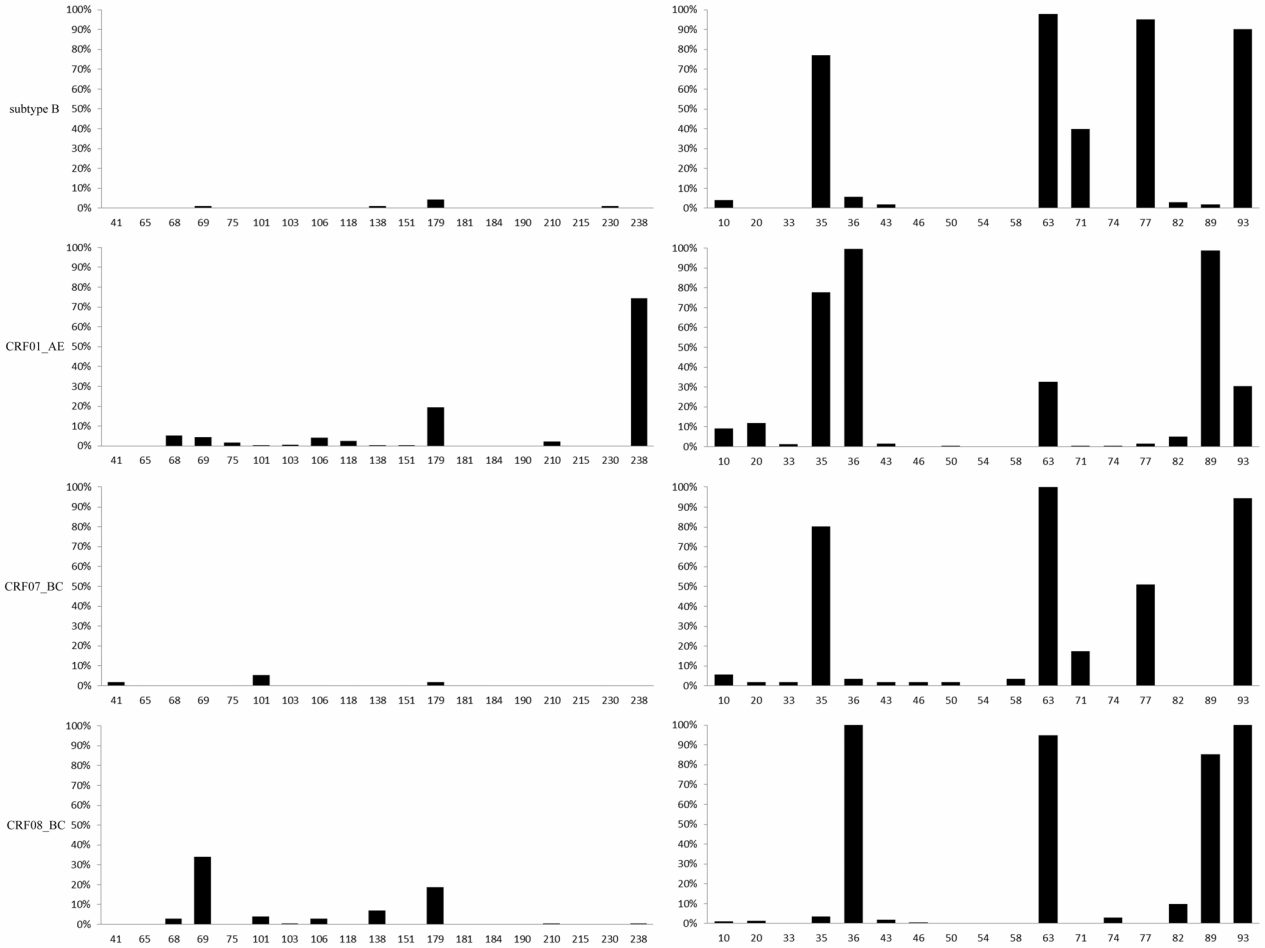
Subtype-specific drug resistance mutation sites genetic background analysis. Note: left part based on RT region and right part based on protease region, horizontal numbers indicate drug resistance mutation sites with significant difference among subtypes(chi-square or fisher's exact test, *p*<0.05), vertical percentage indicate the proportion of mutated amino acids differ from the subtype B consensus sequence.

To compare the polymorphism of genetic background in sites related to drug resistance, sequences obtained from drug-sensitive strains which were isolated from drug-naïve individuals were analyzed. To guarantee that all sequences were obtained from drug-naïve individuals, only sequences generated in our laboratory with a clear clinical background were used. The analysis will be useful to explore the background of drug resistance sites and distinguish polymorphisms from drug resistant mutations. For subtype B, all sites matched the consensus sequence in RT region well except sites 69, 138, 179 and 230, which were present in 1–4% of sequences analyzed. However, amino acids proportion of the other three subtypes were more complicated especially in CRF01_AE and CRF08_BC (Figure 2). Sites 68, 69, 75, 106, 118, 179, 210, 238 in CRF01_AE, sites 41, 101, 179 in CRF07_BC and sites 68, 69, 101, 106, 138,179 in CRF08_BC with obvious higher mutated proportions (2–74%), which should classified as a polymorphism site in respective subtypes. The result of drug resistance mutation sites in protease region was complicated and difficult to understand because so many sites with dominant proportions in drug naïve sensitive sequences. Several sites including 35, 36, 63, 71, 77, 89 and 93 show surprising high mutated amino acid proportions 5–98% in almost all subtypes despite according to the HIVdb Genotypic Resistance Interpretation Algorithm those mutations did not confer to drug resistance due to a small mutation scoring change. For example, site 71 in subtype B accounts for about 40% of mutated amino acids, this proportion apparent higher than that reported in previous published articles for A71T/V only occurred in 2–3% untreated persons and A71I/L were nonpolymorphic mutations that occurred with multiple PIs-related drug resistance mutations. Similar conditions were observed in most other sites suggesting that those mutations occurred and/or were selected during the process of virus replication *in vivo* without exposure to ART. Therefore accurate analysis of amino acids in drug resistance mutation sites among untreated population is not only necessary but helpful to better understand the drug resistance background status of HIV-1 PIs in China and this will provide some useful information for the application of PIs in future to get satisfactory therapic effect.

### Different frequencies of drug-resistance related sites were observed in susceptible strains isolated from treated individuals

**Figure 3:**
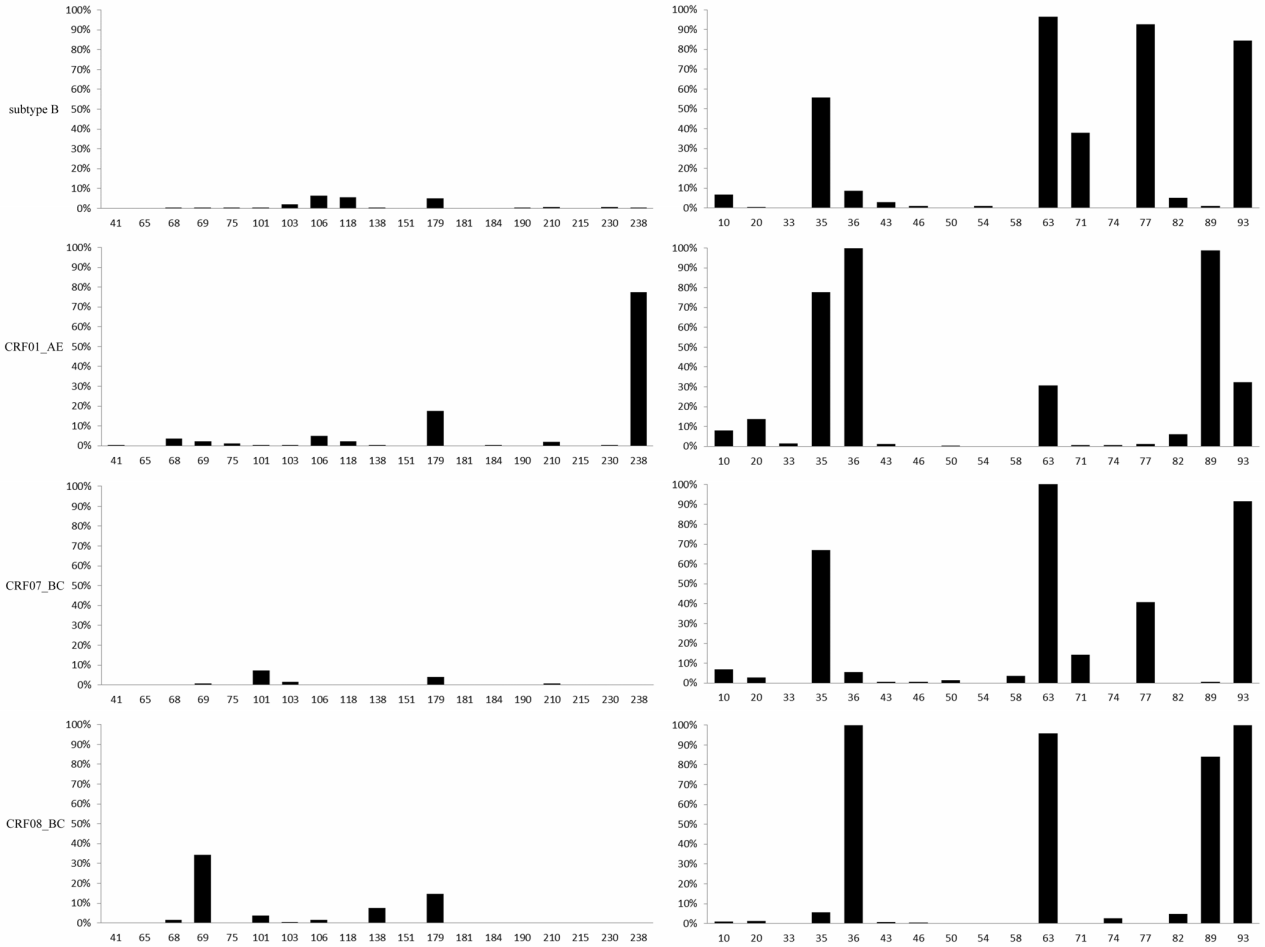
Subtype-specific drug resistance mutation sites analysis based on ART sensitive sequences. Note: left part based on RT region and right part based on protease region, horizontal numbers indicate drug resistance mutation sites with significant difference among subtypes(chi-square or fisher's exact test, *p*<0.05), vertical percentage indicate the proportion of mutated amino acids differ from the subtype B consensus sequence.

Considering polymorphism at sites associated with HIV drug resistance may show different results under drug pressure, the distributions of amino acids at sites related to drug resistance among different HIV strains susceptible to antiretroviral drugs isolated from ART treated individuals were investigated. All sequences were collected during drug resistance annual surveillance and epidemiological study in our laboratory with clear background. More sites with higher proportion of mutated amino acids were observed in RT region in treated population as sites 103, 106, 118 in subtype B, sites 41, 184, 230, 238 in CRF01_AE, sites 69, 103, 210 in CRF07_BC. However, sites associated with drug resistance as 68, 106, and 179 in CRF08_BC show lower proportion of mutated amino acids under drug pressure (Figure 3). Those results suggesting that antiretroviral drugs play certain roles in induce and/or select mutations. Interestingly, the proportions of mutated amino acids in several sites related to drug resistance were lower in protease of respective groups despite little even no exposure to PIs and this further proved that drugs may have effects on HIV genetic mutations even in an indirect way. What we emphasize here is drug resistance mutation sites always compatible with polymorphism sites for the latter usually contains at least two kinds of nucleotides even if encode synonymous amino acid often lead to the spatial structure change greatly and influence the affinity of antiretroviral drugs and their targets cause drug resistance finally.

### Difference at drug-resistance related sites in drug-sensitive strains between treated and untreated patients

Use of drugs always influence the alleles of polymorphism sites in targeting region, which may have effect on the development of drug-resistance. In this study, the amino acid proportions of mutated sites associated with drug resistance of each subtype between the drug naïve and ART treated sensitive sequences were also compared. More mutation sites with higher mutated amino acid proportions were observed in almost all subtypes of the latter group, which reveals that antiretroviral drugs usually induce and/or select several mutations hardly occurred in RT region of drug naïve sequences. Those mutations can be divided into three types based on their functions as follows: i) mutations not associated with drug resistance according to available public published articles so far like K101R, K103R, V106I and V179I; ii) mutations that alone decrease susceptibility and contribute to drug resistance very limited usually work in combination with certain drug resistance mutations like V75I, V179F and L210W; iii) mutations not reported previously for low proportion whose conference to drug resistance are uncertain. What's more impress is that those sites in subtype B also emerged in at least one of other three subtypes as that in drug resistance sequences. In contrast, despite few sites show apparent difference between the drug naïve and treated sensitive groups, most sites in protease region were similar possibly due to the absent use of PIs in China.

## Discussion

In this study, we fulfilled, to our knowledge, the first comprehensive study on amino acid distributions of drug-resistant sites among HIV subtypes prevalent in China, though studies in this areas have been carried up for more than 10 years [17]–[23]. As the dominant subtype prevalent in modern countries, subtype B was studied in more details than other subtypes. Most genetic characterizations of drug-resistant strains were also deduced basing on subtype B strains. However, the most studied subtype B caused only 12% of global infections, while subtype C was responsible for nearly 50% of prevalent infections and 47% of all new infections in the whole world [23]. More and more studies showed that HIV-1 subtype diversity had great effects on the development of HIV drug resistance, data on baseline antiretroviral susceptibility derived from studies of subtype B may not be applicable to non-B subtypes. Several drug resistance mutations were only identified in specific subtypes or showed different prevalence among different subtypes [3]. Furthermore, some strains were showed naturally resistant to antiretroviral drugs, for example, HIV-2 is intrinsically resistant to almost all NNRTIs. So it is urgent to illustrate the influence of HIV-1 subtypes on antiretroviral treatment and drug resistance development, which will be helpful to guide clinic antiretroviral therapy in design treatment strategies basing on subtype identification.

The subtype B virus is prevalent in China and the sequences included in this study were mainly Thai B, which was genetically distinct from the subtype B that dominant in the US and Europe as European B. Differences between Thai B and European B are mainly based on the C2-V3 region of the *env* gene, especially the motif sequences at crown of the V3 loop. In European B variants, the typical sequence is GIHIGPGRAFYTTG, while in Thai B the dominant pattern is SIPLGPGQAWYTTQ [24]–[26]. Considering that ART drugs targets were encoded by *pol* gene, which is more conservative and stable during viral replication *in vivo* for structure and function maintenance, so it's acceptable that the drug resistance of Thai B variants can be evaluated by the Stanford HIV drug resistance database which are based on data mainly from European B. Furthermore, despite CRF07_BC is a prevalent virus in China, few drug resistant sequences were collected. CRF07_BC is mainly epidemic in IDUs and MSM, for which population HAART and HIV drug resistance surveillance were hard to be fulfilled. With the scaling up of free therapy in China, more patients infected with CRF07_BC will be included into therapy and this may explain why most CRF07_BC variants included were drug-susceptible. In the future, our group will cooperate with first line workers to reinforce the drug resistance surveillance of CRF07_BC.

Mutation from a wildtype to a drug resistant codon could be influenced by the number and type of nucleotide(s) [16]. For example, it was proved that transitions (the replacement of a purine by another purine or a pyrimidine by another pyrimidine) are for steric reasons occurring on average 2.5 times more frequently than transversions (the replacement of a purine by a pyrimidine and vice versa) [27]. Furthermore, the number of hydrogen bonds between paired nucleotides may also affect the ratio of mutation. Since only two hydrogen bonds were identified between AT basepair while three hydrogen bonds were between GC basepair, it is reasonable to find more mutations at AT polymorphism sites. High level of polymorphism at drug-resistance related sites among different subtypes definitely affects mutation sites or patters. In this study, obvious disparities among HIV-1 subtypes were observed at drug-resistance related sites with different genetic background. Therefore, polymorphisms at drug resistance related sites may contributed differently to the occurrence of HIV drug resistance and should be given more concerns during the surveillance and design of antiretroviral therapeutic schedules.

The amino acid distributions of drug-resistant mutations sites showed significant difference among HIV-1 subtypes prevalent in China. However, all those sites that determined basing on subtype B strains could be found in at least one non-B virus, which was in accordance with previously published research. The most prevalent drug-resistant mutation sites were K103, Y181 and M184 in all subtypes, suggesting that the HAART regimens may play important roles in selecting the drug-resistant mutations irrespective of HIV subtypes. K103 and Y181 associated drug resistance mutations conferred high level resistance to NVP and EFV, while M184 associated drug resistance mutation cause high level resistance to 3TC and low level resistance to ddI and abacavir (ABC). Other high frequency (more than 10%) drug resistance mutation sites as M41, T69, K101, V106, V179, G190, L210 and T215 were also shared among subtype B, CRF01_AE and CRF08_BC. Among of them, M41, T69, L210 and T215 make up the thymidine analogue-associated mutations (TAMs) and confer high level resistance or low susceptibility to AZT, d4T, ddI, ABC and 3TC; K101, V106 and G190 were NNRTI-associated mutations contribute resistance to NVP, EFV and ETR. Most of those drugs were included in first and/or second line regimens used in China. Come to the conclusion that mutations listed above were all selected under drug pressure except V106 in subtype B, V179 and K238 in CRF01_AE and T69 and V179 in CRF08_BC due to little difference of prevalence between susceptible and resistance groups and should be owing to polymorphism sites in corresponding subtypes. Different to RT region, high level of polymorphism at drug-resistance related sites were found in protease region. It probably correlates to long time selection and viral evolution *in vivo*, so host races, geography distribution may have influence. Some sites (for example, M46L in CRF01_AE) were demonstrated in high level in drug-naïve population and should be distinguished for further study and considered in clinic therapy.

Several factors may affect the development of HIV-1 drug resistance *in vivo*: First, High speed of HIV replication and error-prone reproduction of reverse transcriptase. In untreated patients, the total number of productively infected cells in lymphoid tissue estimated to be approximately 10^7^ to 10^8^ and can produce no less than 10^10^ viruses per day [28], [29]. Lack of proofreading activity of reverse transcriptase leads to high rate of mismatch introducing on average one mutation for each viral genome transcribed [23], [30], [31]. Second, genetic barriers conferred by genetic background. Virus single mutation could quickly arise drug resistance to NVP, 3TC or emtricitabine(FTC), but other NRTIs need more mutations which conferred higher genetic barriers to generate drug resistance [32]. Third, single nucleotide polymorphism sites (SNPs) in host genome. It was proved that host genetic polymorphism in some genes could influence the absorption, distribution, metabolism and excretion of certain drugs, and further affect drug concentration in blood, which will lead to changing of effective pressure and production of drug resistance [33], [34]. For example, SNPs in cytochrome P450 and the ATP-binding cassette family genes can greatly affect the blood concentration of ART drugs and further put impact on treatment effectiveness [35]–[42]. Other behavioral factors, such as good adherence, were also necessary to ensure success ART treatment, which had been absolutely confirmed [43].

In conclusion, we analyzed the distribution of amino acids at drug-resistant sites among HIV-1 subtypes prevalent in China, including subtype B, CRF01_AE, CRF07_BC and CRF08_BC. Although almost all mutations in subtype B were found at least in one non-B virus, different distributions of drug resistance mutation sites among HIV-1 subtypes were identified. The results hint that HIV-1 drug employment should be individualized, drugs with high genetic barriers should be given more consideration. The detailed data demonstrated in this study will be helpful for design regimens compatible for Chinese according to the prevalent subtypes.

## Supporting Information

File S1 (DOC)
