# Supplementary material for: Different Frequencies of Drug Resistance Mutations among HIV-1 Subtypes Circulating in China: A Comprehensive Study
Source: PLoS One. 2014 Mar 24;9(3):e91803. doi: 10.1371/journal.pone.0091803 (PMC3963863; doi:10.1371/journal.pone.0091803)
Supplement: File S1 — (DOC) [file pone.0091803.s001.doc]

**Different Frequencies of Drug Resistance Mutations among HIV-1 Subtypes Circulating in China: A Comprehensive Study**

**ABSTRACT**

The increase in HIV drug resistance is threatening HIV treatment options in China. Although HIV drug-resistant mutations have been identified and addressed, most of these were based on HIV subtype B. Genetic and HIV subtypes differences are critical to the development of drug-resistant mutations. In China, the main epidemic subtypes are CRF07_BC, CRF08_BC, Thai B and CRF01_AE, so the ability to manage drug resistance mutations of those subtypes may inform regimen selection in China. Here, we studied the amino acid distribution differences among sites related to HIV drug resistance and compared these among subtypes B, CRF01_AE, CRF07_BC and CRF08_BC strains prevalent in China. The sequence amino acid composition of different subtypes obtained from untreated and treated individuals were also compared. Amino acids proportions of 19 sites in RT among subtypes B, CRF01_AE and CRF08_BC were significantly different with respect to drug resistance groups (Chi-square test, *p*<0.05). Genetic barrier analysis revealed that sites 69, 138, 181, 215 and 238 were significantly different among subtypes (Kruskal Wallis test, *p*<0.05). All subtypes had three sites bearing the greatest drug resistance in common: 103, 181 and 184. Many drug-resistant HIV proteases were detected in almost all subtypes in drug-naïve patients. This is the first comprehensive study in China to examine drug resistance development among different HIV subtypes and these data will lay a foundation for HIV treatment regimen design and improve HIV therapy in China.

**Introduction**

Three decades have passed since the human immunodeficiency virus (HIV) was confirmed to be the pathogenic mechanism of acquired immunodeficiency syndrome (AIDS) . Still, HIV remains an established pandemic with enormous social and economic costs. Highly active antiretroviral therapy (HAART) can suppress HIV replication *in vivo* and prolonged and improving the quality of life of HIV-infected persons, but drug resistance still plagues antiretroviral therapy (ART) failure .

HIV genetic diversity contributes to resistance, and HIV-1 and HIV-2 have been identified. HIV-1 is responsible for the global pandemic, comprising most HIV isolates, and HIV-2 is restricted to western and central Africa, accounting for no more than 2% of all HIV infections . HIV-1 consists of M, N, O and P groups, and the M group affects the most people. The M group also has nine pure subtypes (A to D, F to H, J and K), more than 50 circulating recombinant forms (CRFs) and many unique recombinant forms (URFs) . So far, antiretroviral drugs have been designed based on subtype B, the major subtype prevalent in the US and Western Europe which contributes to ~10% of all HIV infection . Numerous genetic polymorphisms have been confirmed among HIV sequences of different subtypes. These may contribute to different drug resistance mutations among HIV subtypes and understanding this will allow us to individualize antiretroviral therapy.

Among the 9 antiretroviral drugs provided by the Chinese government, zidovudine (AZT), lamivudine (3TC), stavudine (d4T), didanosine (ddI) and tenofovir (TDF) are nucleoside reverse transcriptase inhibitors (NRTIs). Efavirenz (EFV) and nevirapine (NVP) are non-nucleoside reverse transcriptase inhibitors (NNRTIs), Indinavir/r(IDV/r) and lopinavir/r (LPV/r) are protease inhibitors (PIs) . First line ART regimens are usually composed of two NRTIs (two of these: d4T, AZT, or 3TC) and one NNRTI (NVP or EFV). Protease inhibitors are typically reserved for people who are unsuccessful with ART or for pregnant women and those who are co-infected with tuberculosis, HBV, and other diseases . Generally, viral subtypes at a population level are not considered during ART regimen selection despite HIV-1 subtype differences which may variously confer drug resistance.

Here, HIV-1 strains from the most prevalent subtypes in China, including subtypes B, CRF01_AE, CRF07_BC and CRF08_BC, were investigated to study drug resistant mutations among them and to correlate mutations with subtypes for the purpose of improving antiretroviral treatment.

**Materials and methods**

**HIV-1 sequences**

The Pol region, especially the region coding protease and the start 720 base pairs of reverse transcriptase (RT), is used for HIV genotype drug-resistance tests. Three HIV-1 sequences within that region were included in this study: i) sequences fragments downloaded from the Los Alamos HIV sequences database ([http://hiv-web.lanl.gov](http://hiv-web.lanl.gov/)). Problematic sequences were excluded and only one sequence was selected per patient; ii) sequences generated in our laboratory for annual drug resistance surveillances and epidemiological analysis in the past 3 years were also included. All sequences were produced by nest RT-PCR with plasma viral RNA as template, and then sequenced with a pyrosequencing method; iii) sequences provided by the AIDS Care Center of the Yunnan Infectious Disease Hospital during drug resistance surveillance in 2012. To ensure data quality, sequences containing stop codons and individual resistance codons with more than 2 ambiguous base per nucleotide position or those with 2 ambiguous bases, which may have come from contamination in RT-PCR or bad sequencing, were excluded from the analysis.

**Sequences subtyping and drug resistance evaluation**

Subtypes of HIV sequences from the database were identified and downloaded. Sequences generated in our laboratory and those provided by the AIDS Care Center of the Yunnan Infectious Disease Hospital were subtyped with the online REGA HIV-1 subtyping tool 3.0 ([http://www.bioafrica.net](http://www.bioafrica.net/)), which incorporates both phylogenetic and boot scanning methods in an automated process to identify subtypes, CRFs and URFs of query sequences . MEGA 5.1 was used to perform phylogenetic analysis to identify sequence subtypes which could not be determined with REGA. All sequences of subtype B, CRF01_AE, CRF07_BC and CRF08_BC were included in the drug resistance evaluation, which was performed online using the HIV drug resistance database ([http://www.hivdb.stanford.edu](http://www.hivdb.stanford.edu/)).

Sequences were sorted into groups based on subtypes and drug resistance. The resistant sequence group was comprised of all four subtype sequences with any major and/or minor drug resistant mutation(s). The susceptible sequence groups were composed of sequences containing no drug-resistant mutations, and sequences were further subdivided into three small groups according to ART conditions: a) totally susceptible group, containing all sequences; b) a drug naïve susceptible group, containing sensitive sequences isolated from patients never exposed to ART; and c) an ART susceptible group, containing sensitive sequences isolated from patients who were given ART.

**Drug resistant mutations**

Drug resistant mutations were defined as differences from the consensus B amino acid sequence and selected based on the HIV drug resistance database and published data including all listed major, minor and other mutations which are polymorphism sites or those which occur in combination with mutations obviously influence drug resistance. A polymorphism is defined as a mutation that occurs in at least 1% of a population not exposed to selective drug pressure . Drug-resistance mutation sites are selected based on drugs used in China as follows: NRTI-resistance related sites 40, **41**, 44, 62, **65**, 66, **67**, 68, **69**, **70**, 71, **74**, 75, 77, **115**, 116, 118, **151**, **184**, **210**, **215** and **219**; NNRTI-resistance related sites 98, **100**, **101**, **103**, **106**, 108, **138**, 179, **181**, **188**, **190**, 225, 227, **230**, 236 and 238; and PI-resistance related sites 10, 11, 20, 23, 24, **30**, **32**, 33, 35, 36, 43, **46**, **47**, **48**, **50**, 53, **54**, 58, 63, 71, 73, 74, **76**, 77, **82**, 83, **84**, 85, **88**, 89, **90** and 93. Bolded numbers are major drug-resistance mutation sites that can lead to drug resistance in the absence of other mutations. Minor drug-resistance mutation sites cannot cause drug resistance alone; they occur with major drug resistance mutations to decrease virus susceptibility and/or increase drug resistance .

**Statistical analysis**

Sequences were aligned with BioEdit v7.0 and manually edited before translating into amino acids. The frequencies of amino acids at every drug resistance site were calculated. To analyze the differently distribution of amino acids among different subtypes, chi-square or fisher’s exact test were fulfilled with SPSS v17.0 software after excluding sequences containing amino acids like - (means bases absence or not been tested) and x (means existing at least one ambiguous base causing a translated amino acid was unknown) from the analysis. If positive results were observed, a Kruskal Wallis test was performed to ascertain whether the difference was due to genetic barriers of codons.

**Results**

**HIV-1 sequences overview**

A total of 3,624 sequences were downloaded from an HIV sequence database, and 1,483 sequences were generated in our laboratory; 335 sequences were provided by the AIDS Care Center of Yunnan Infectious Disease Hospital. There were 1984 subtype B, 1696 CRF01_AE, 502 CRF07_BC and 924 CRF08_BC sequences included in this study. For analytic convenience, comparisons of nucleotide(s) insertions and deletions to subtype B consensus sequences were omitted. The whole sequence was separated into a protease region (amino acids: 1–99) and an RT region (amino acids: 1–240; Table S1).

**of drug-resistant mutations among subtypes B, CRF01_AE and CRF08_BC**

To analyze the distribution of drug-resistant mutations among different HIV subtypes, 38 sites in the RT region were analyzed (PI-resistant mutations were not analyzed; only 73 PI-resistant sequences were obtained: CRF07_BC and CRF08_BC which had 4 and 7 sequences, respectively). All drug-resistant mutations identified based on subtype B were found in at least one of the other two subtypes. Significantly different distributions were observed in 19 sites among subtypes B, CRF01_AE and CRF08_BC (CRF07_BC was not considered due to a small sample size, Chi-square or Fisher’s exact test, *p*<0.05). Among of them, three sites, including 103, 181 and 184, were most prevalent (>20%) in all three subtypes. Drug resistance mutation distributions were different among subtypes: sites 41, 69, 101, 106, 179, 190, 210 and 215 were dominant in subtype B, whereas sites 68, 69, 75, 101, 106, 179, 190, 210, 215 and 238 in CRF01_AE and sites 41, 69, 101, 179 and 190 in CRF08_BC were more prevalent than 10% (Figure s1). Further analyses of codons of those sites indicate that synonymous amino acid mutations caused by single base mutations existed in all sites except sites 41, 184 and 230. Nonsynonymous mutations of amino acids from 1 to 4 in different sites caused by different bases mutations existed in almost all sites. Typical mutations such as M41I, K65N, V75AS, K101P, E138K, Q151L, V179F and G190EQ were not observed in this study. Genetic barriers, defined as the number of base changes for a virus to develop resistance and escape selection pressure from drugs , are thought to contribute to the development of HIV drug resistance. In this study, 5 sites (69, 138, 181, 215 and 238) among the 19 sites analyzed had significant differences among subtypes (Kruskal Wallis test, *p*<0.05) (Table S2). This observation may explain why drug resistance develops at a similar rate during antiretroviral therapy in China irrespective of subtype prevalence. Few minor drug-resistance mutation sites such as 179 in subtype B, 75, 179, 238 in CRF01_AE and 179 in CRF08_BC account for the majority of corresponding subtypes compared to major drug-resistance mutation sites that have dominant roles.

**Polymorphisms related to drug-resistance among subtypes in drug-naïve individuals**

To compare polymorphisms in sites related to drug resistance, sequences obtained from drug-sensitive strains isolated from drug-naïve individuals were analyzed. To guarantee that all sequences were obtained from drug-naïve individuals, only sequences generated in our laboratory with a clear clinical background were used. Such an analysis will enable us to study drug resistance sites and distinguish polymorphisms from drug resistant mutations. For subtype B, all sites matched the consensus sequence in the RT region except sites 69, 138, 179 and 230, which were present in 1–4% of the sequences analyzed. However, the amino acids of the other three subtypes were more complicated, especially in CRF01_AE and CRF08_BC (Figure s2). Sites 68, 69, 75, 106, 118, 179, 210, 238 in CRF01_AE, site 41, 101, 179 in CRF07_BC and sites 68, 69, 101, 106, 138,179 in CRF08_BC had more mutations (2-74%), which should classified as a polymorphism site in respective subtypes. Drug resistance mutation sites in the protease region were complicated; many sites had dominant proportions in drug-naïve sensitive sequences. Several sites including 35, 36, 63, 71, 77, 89 and 93 had many mutated amino acids (5–98% in almost all subtypes according to the HIVdb Genotypic Resistance Interpretation Algorithm which indicated that those mutations did not confer to drug resistance due to a small mutation scoring change. For example, site 71 in subtype B accounts for ~40% of mutated amino acids, a higher proportion than that reported in previous reports for A71T/V (2–3% occurred in untreated persons; A71I/L were nonpolymorphic mutations that occurred with multiple PI-related drug resistance mutations). Similar conditions were observed in most other sites, suggesting that those mutations occurred and/or were selected during the process of virus replication *in vivo* without ART exposure. Therefore accurate amino acid analysis in drug-resistance mutation sites among untreated populations is not only necessary but also permits understanding the drug resistance background status of HIV-1 PIs in China. This may permit individualized HIV therapy and better patient outcomes.

**Frequencies of drug-resistance related sites observed in susceptible strains isolated from treated individuals**

Polymorphisms at sites associated with HIV drug resistance behave differently with drug exposure. Amino acid distributions at sites related to drug resistance among different HIV strains susceptible to antiretroviral drugs were isolated from ART-treated individuals were investigated. All sequences were collected during a drug resistance annual surveillance and epidemiological study in our laboratory. Sites with more mutated amino acids were observed in the RT region in treated populations (sites 103, 106, 118 in subtype B, site 41, 184, 230, 238 in CRF01_AE, site 69, 103, 210 in CRF07_BC). However, sites associated with drug resistance (68, 106, and 179 in CRF08_BC) had fewer mutated amino acids under drug pressure (Figure s3). Thus, antiretroviral drugs may induce and/or select for mutations. Interestingly, mutated amino acids in several sites related to drug resistance were lower in the protease of respective groups despite little or no exposure to PIs. So, HIV drugs may have effects on HIV genetic mutations indirectly. Drug resistance mutation sites are always compatible with polymorphism sites and they usually contains at least two kinds of nucleotides even if they encode synonymous amino acids and cause spatial structural changes that influence the affinity of antiretroviral drugs and their targets to cause resistance.

**Differences in drug-resistance related sites in drug-sensitive strains between treated and untreated patients**

Drug treatment affects polymorphism allele sites in targeting regions, which may cause drug-resistance. Mutated amino acids associated with drug resistance of each HIV subtype in drug naïve and ART-treated sensitive sequences revealed more mutation sites with more mutated amino acids in almost all HIV subtypes after ART, suggesting that antiretroviral drugs induce and/or select several mutations that do not occur in the RT region of drug naïve sequences. These mutations are of three types based on their functions: i) mutations not associated with drug resistance according to the literature (K101R, K103R, V106I and V179I); ii) mutations that decrease susceptibility and contribute to drug resistance usually in combination with drug resistance mutations (V75I, V179F and L210W); iii) mutations not reported previously or those which are uncertain. Sites in subtype B also emerged in at least one of the other three subtypes as drug resistance sequences. In contrast, few sites were different between drug naïve and treated sensitive groups, but most sites in the protease region were similar likely due to the infrequent use of PIs in China.

**Discussion**

This represents, to our knowledge, the first comprehensive study of amino acid distribution within drug-resistant sites among HIV subtypes prevalent in China . Subtype B, the most prevalent subtype has been under intense scrutiny and most genetic characterizations of drug-resistant strains have been deduced using subtype B strains. However, subtype B causes only 12% of global infections, and subtype C is responsible for nearly 50% of existing and 47% of all new HIV globally . Increasing evidence suggests that HIV-1 subtype diversity influences HIV drug resistance, and data for antiretroviral susceptibility derived subtype B studies may not apply to non-B subtypes. Several drug resistance mutations have been identified in specific subtypes or were unique with respect to prevalence among different subtypes . Furthermore, some HIV strains are naturally resistant to antiretroviral drugs. HIV-2 is intrinsically resistant to almost all NNRTIs. Thus, understanding how HIV-1 subtypes affect antiretroviral treatment and drug resistance development will guide antiretroviral treatment strategies using subtype identification.

Mutations from a wild type to a drug-resistant codon could be influenced by the number and type of nucleotide(s) . For example, transitions (purine-purine or pyrimidine-pyrimidine replacements) occur due to steric issues 2.5 times more frequently than transversions (purine-pyrimidine or pyrimidine-purine replacements) . Furthermore, the number of hydrogen bonds between paired nucleotides may affect the mutation ratio. Because only two hydrogen bonds have been identified between AT base pairs and three hydrogen bonds exist GC base pairs, more mutations would be expected at AT polymorphic sites. Increased polymorphism at drug-resistance related sites among different HIV subtypes affects mutation sites or patterns. Here, we report gene-related disparities among HIV-1 subtypes within drug-resistance related sites, and we suggest that these polymorphisms may contribute uniquely to HIV drug resistance. Thus, they should be considered during surveillance and design of antiretroviral therapeutic schedules.

Amino acid distributions of drug-resistant mutation sites were significantly different among HIV-1 subtypes prevalent in China. However, all sites found in subtype B strains were also observed in at least one non-B virus, a finding that agreed with the literature. The most prevalent drug-resistant mutation sites were K103, Y181 and M184 in all HIV subtypes, suggesting that HAART regimens are key to selecting drug-resistant mutations irrespective of HIV subtype. K103- and Y181-associated drug resistance mutations were highly resistant to NVP and EFV, whereas M184-associated drug resistance mutations caused resistance to 3TC and low level resistance to ddI and abacavir (ABC). Other high frequency (>10%) drug-resistance mutation sites M41, T69, K101, V106, V179, G190, L210 and T215 were also shared among subtype B, CRF01_AE and CRF08_BC. Among of them, M41, T69, L210 and T215 comprise thymidine analogue-associated mutations (TAMs) and confer high-level resistance or low susceptibility to AZT, d4T, ddI, ABC and 3TC; K101, V106 and G190 were NNRTI-associated mutations contribute resistance to NVP, EFV and ETR. Most of those drugs were first and/or second line regimens used in China. Thus, the mutations studied here were all selected under drug pressure except V106 in subtype B, V179 and K238 in CRF01_AE and T69 and V179 in CRF08_BC due to little differences in prevalence between susceptible and resistant groups attributed to polymorphisms in corresponding subtypes. Also, polymorphisms at drug-resistance related sites were found in the protease region, likely correlating to selection over time and viral evolution. Thus, race and geographic distribution may influence these resistances. Some sites (for example, M46L in CRF01_AE) were confirmed in drug-naïve populations and warrant further study.

Several factors may affect the development of HIV-1 drug resistance. First, rapid and error-prone HIV replication of reverse transcriptase may occur. In untreated patients, infected cells in lymphoid tissue are estimated to be ~10^7^ to 10^8^, capable of producing more than 10^10^ viruses per day . Lack of reverse transcriptase proofreading activity leads to more mismatches and the introduction of one mutation for each viral genome transcribed . Second, genetic barriers may exist. Viral single mutations could quickly offer drug resistance to NVP, 3TC or emtricitabine (FTC), but other NRTIs require more mutations to confer higher genetic barriers to generate drug resistance . Third, single nucleotide polymorphism sites (SNPs) in the host genome have been reported to influence the absorption, distribution, metabolism and excretion of certain drugs, and alter serum drug concentrations, changing the effective pressure and production of drug resistance . For example, SNPs in cytochrome P450 and the ATP-binding cassette family of genes can alter concentrations of ART drugs and decrease treatment effectiveness . Other behavioral factors, such as patient compliance is also necessary to ensure ART treatment success .

In conclusion, we analyzed the distribution of amino acids at drug-resistant sites among HIV-1 subtypes prevalent in China, including subtypes B, CRF01_AE, CRF07_BC and CRF08_BC. Although almost all mutations in subtype B were found at least in one non-B virus, different distributions of drug-resistance mutation sites among HIV-1 subtypes were identified. Our data show that HIV-1 drug choice should be individualized, and genetic barriers should be given consideration. Thus our data should improve the design of ART dosing regimens for Chinese populations based on prevalent HIV subtypes.

**Acknowledgements**

The authors thank Yang Shaomin for providing sequences from the AIDS Care Center of the Yunnan Infectious Disease Hospital, and Zhuang Daomin, Li Tianyi, Liu Siyang and Wang Xiaolin for helpful discussions.

**Figure Legends**

Figure S1 Subtype-specific drug-resistance mutation sites analysis

Note: The x-axis indicates drug-resistance mutation sites in the RT region with significant differences among subtypes. The y-axis indicates the proportion of mutated amino acids that differ from the subtype B consensus sequence. Black columns depict amino acid distributions with significant differences among subtypes (Chi-square or Fisher’s exact test, *p*<0.05). White columns depict mutated amino acid distributions and genetic barriers with significant differences among subtypes (Kruskal Wallis test, *p*<0.05).

Figure S2 Subtype-specific drug-resistance mutation sites genetic background analysis

Note: left are based on RT region and right are based on protease region, The x-axis indicates drug-resistance mutation sites with significant differences among subtypes (Chi-square or Fisher`s exact test, *p*<0.05). The y-axis indicates the proportion of mutated amino acids that differ from the subtype B consensus sequence.

Figure S3 Subtype-specific drug-resistance mutation sites analysis based on ART sensitive sequences. Note: left are based on the RT region and right are based on the protease region. The x-axis indicates drug-resistance mutation sites with significant differences among subtypes (Chi-square or Fisher`s exact test, *p*<0.05). The y-axis indicates the proportion of mutated amino acids that differ from the subtype B consensus sequence.

Figure S1


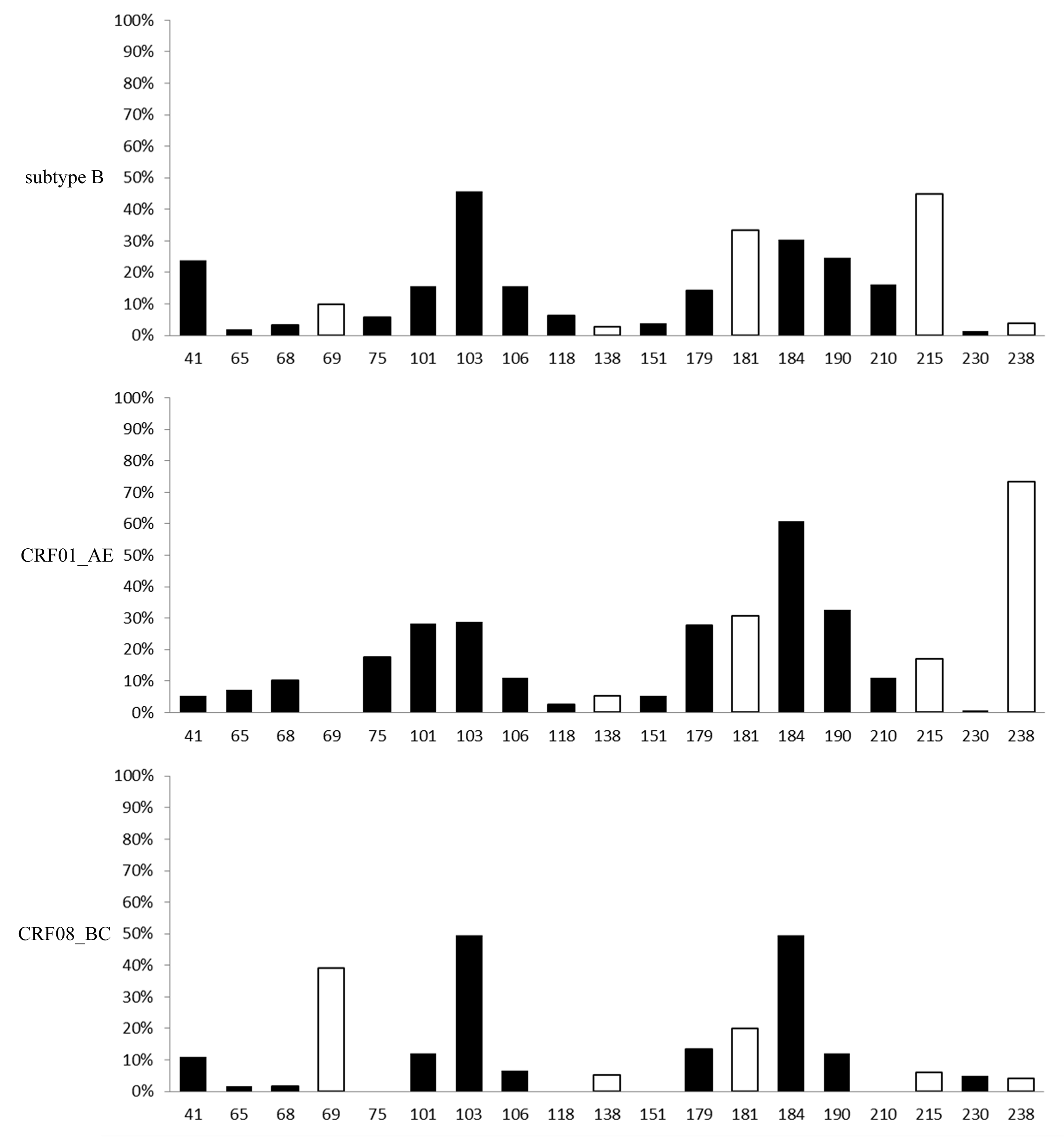


Figure S2
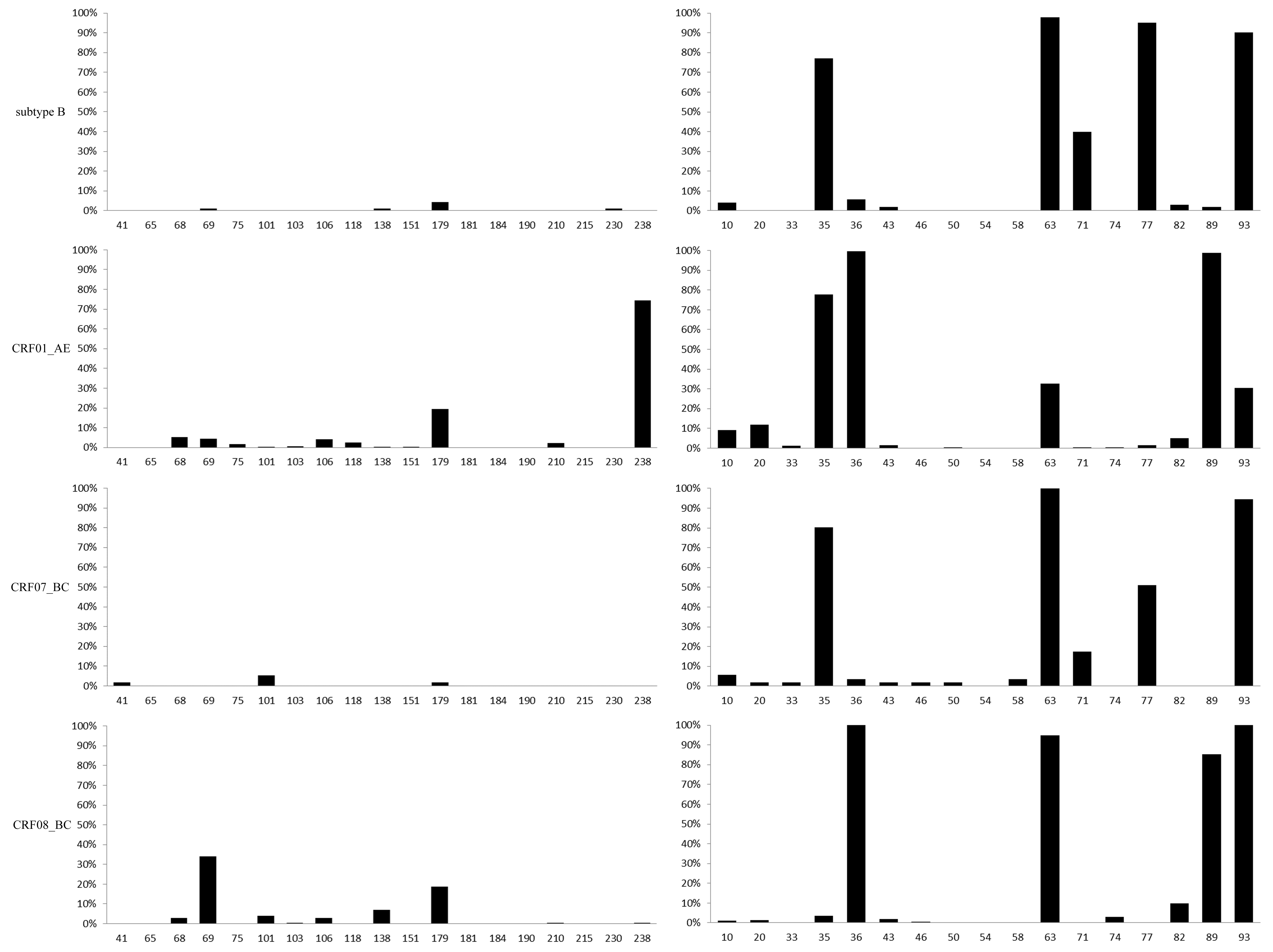


Figure S3


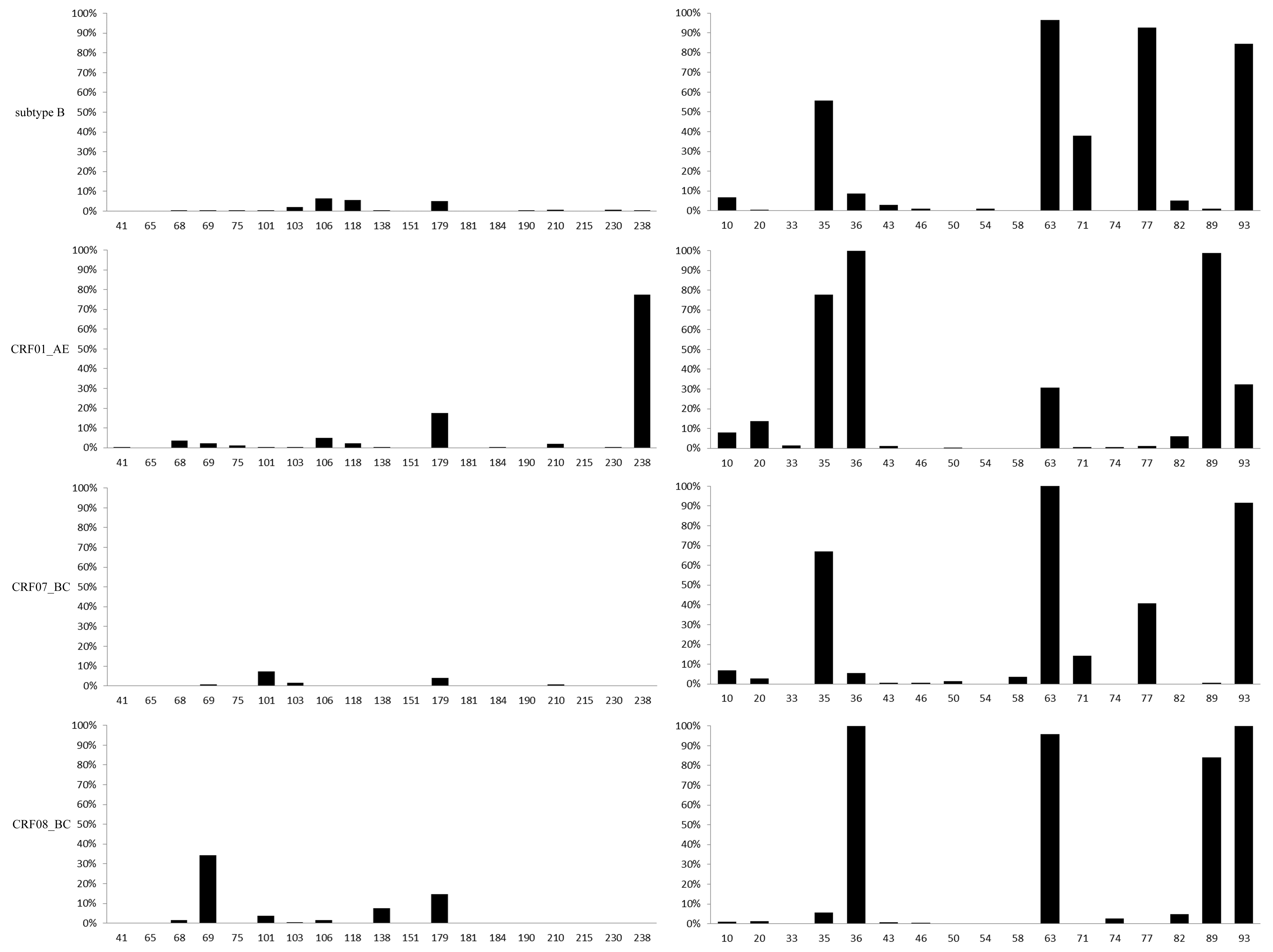


**Table S1** Sequence summary

| **Subtype** | **resistance** | |  | **total sensitive** | |  | **drug naïve sensitive** | |  | **ART sensitive** | |
| --- | --- | --- | --- | --- | --- | --- | --- | --- | --- | --- | --- |
|  | **Protease** | **RT** |  | **Protease** | **RT** |  | **Protease** | **RT** |  | **Protease** | **RT** |
| subtype B | 32 | 700 |  | 1422 | 1207 |  | 104 | 99 |  | 175 | 398 |
| CRF01_AE | 30 | 152 |  | 1659 | 1725 |  | 426 | 421 |  | 549 | 550 |
| CRF07_BC | 4 | 27 |  | 441 | 428 |  | 57 | 57 |  | 145 | 126 |
| CRF08_BC | 7 | 120 |  | 940 | 804 |  | 206 | 202 |  | 590 | 484 |

**Table S2** prevalence of wild type codons and genetic barriers of drug-resistance mutation sites

| position | substitution | wt codon | WT codon proportion (%) | | | rt codon | required mutation | *p*^1^ | *p*^2^ |
| --- | --- | --- | --- | --- | --- | --- | --- | --- | --- |
|  |  |  | B (700) | 01_AE (152) | 08_BC (120) |  |  |  |  |
| 41 | M41L | ATG | 74.1 | 94.7 | 87.5 | CTG/TTG | 1 tv | <0.001 | 0.136 |
| 65 | K65R | AAA | 94.2 | 90.8 | 2.5 | AGA | 1 ts | 0.006 | 1.000 |
|  |  | AAG | 3.3 | 1.3 | 95.0 | AGG | 1 ts |  |  |
| 68 | S68G | AGT | 94.3 | 9.2 | 90.8 | GGT | 1 ts | 0.014 | 1.000 |
|  | S68N | AGC | 1.6 | 77.0 | 3.3 | AAT | 1 ts |  |  |
| 69 | T69N | ACT | 87.9 | 11.2 | 58.3 | AAT/AAC | 1 tv | <0.001 | <0.001 |
|  | T69S | ACC | —- | 73.0 | — | AGT | 1 tv |  |  |
|  | T69D |  |  |  |  | GAT | 1 ts, 1 tv |  |  |
| 75 | V75I | GTA | 92.9 | 69.1 | 97.5 | ATA | 1 ts | <0.001 | 0.498 |
|  | V75M | GTG | — | 10.5 | 0.8 | ATG | 1 ts |  |  |
|  | V75T |  |  |  |  | ACA | 2 ts |  |  |
| 101 | K101E | AAA | 81.3 | 64.5 | 80.8 | GAA | 1 ts | 0.009 | 0.116 |
|  | K101Q | AAG | 1.7 | 3.9 | 3.3 | CAA | 1 tv |  |  |
|  | K101R |  |  |  |  | AGA | 1 ts |  |  |
| 103 | K103N | AAA | 50.4 | 66.4 | 38.3 | AAC/AAT | 1 tv | <0.001 | 0.627 |
|  | K103R | AAG | 1.4 | 2.0 | — | AGA | 1 ts |  |  |
|  | K103S |  |  |  |  | AGC | 1 ts, 1 tv |  |  |
| 106 | V106I | GTA | 80.5 | 70.4 | 90.0 | ATA | 1 ts | 0.013 | 0.088 |
|  | V106A | GTG | 1.6 | 12.5 | 1.7 | GCA | 1 ts |  |  |
|  | V106M |  |  |  |  | ATG | 1 ts |  |  |
| 118 | V118I | GTT | 27.0 | 82.9 | 97.5 | ATT | 1 ts | 0.079 | 0.464 |
|  |  | GTC | 63.2 | 9.2 | 0.8 | ATC | 1 ts |  |  |
|  |  | GTA | 1.4 | 1.3 | 0.8 | ATA | 1 ts |  |  |
| 138 | E138Q | GAG | 93.8 | 91.4 | 87.5 | CAG | 1 tv | 0.028 | <0.001 |
|  |  | GAA | 2.9 | 2.0 | 4.2 | CCG | 2 tv |  |  |
| 151 | Q151M | CAG | 92.5 | 86.8 | 94.2 | ATG | 2 tv | 0.025 | — |
|  |  | CAA | 2.4 | 5.9 | 2.5 | ATG | 2tv, 1 ts |  |  |
| 179 | V179I | GTT | 76.7 | 63.2 | 77.5 | ATT | 1 ts | 0.002 | 0.840 |
|  | V179D | GTG | 3.3 | 2.0 | — | GAT | 1 tv |  |  |
|  | V179E | GTC | 2.9 | 4.6 | 3.3 | GAA/GAG | 2 tv/1 tv |  |  |
|  | V179T |  |  |  |  | ACT | 2 ts |  |  |
| 181 | Y181C | TAT | 64.6 | 65.1 | 76.7 | TGT/TGC | 1 ts | 0.029 | 0.012 |
|  | Y181V | TAC | 1.1 | 2.0 | — | GTT | 1 ts, 1 tv |  |  |
|  | Y181I |  |  |  |  | ATT | 2 tv |  |  |
| 184 | M184V | ATG | 68.7 | 37.5 | 50 | GTG/GTA | 1 ts/2 ts | <0.001 | 0.823 |
|  | M184I |  |  |  |  | ATA | 1 ts |  |  |
| 190 | G190A | GGA | 71.6 | 57.2 | 75.0 | GCA | 1 tv | 0.058 | 0.101 |
|  | G190S | GGC | 1.9 | 3.3 | 6.7 | AGC | 1 ts |  |  |
|  |  | GGG | — | — | 2.5 | GCA/AGC | 1 tv/1 ts |  |  |
| 210 | L210W | TTG | 74.8 | 77.6 | 1.7 | TGG | 1 tv | <0.001 | 1.000 |
|  | L210S | TTA | 6.5 | 7.2 | 96.7 | TCG | 1 ts |  |  |
|  |  | CTA | — |  | 0.8 | TGG/TCG | 1 tv/1 ts |  |  |
| 215 | T215Y | ACC | 52.8 | — | 88.3 | TAC/TAT | 2 tv | <0.001 | <0.001 |
|  | T215F | ACT | — | 78.3 | — | TTC/TTT | 1 ts, 1 tv |  |  |
| 230 | M230L | ATG | 98.6 | 98.7 | 94.2 | CTG/TTG | 1 tv | 0.038 | 1.000 |
| 238 | K238T | AAA | 94.0 | 25.0 | 89.2 | ACA | 1 tv | <0.001 | <0.001 |
|  | K238N | AAG | 0.9 | 0.7 | 4.2 | AAC | 1 tv |  |  |
|  | K238R |  |  |  |  | AGA | 1 ts |  |  |

Abbreviations: wt – wild type; rt – resistance type; ts – transition; tv – transversion; underlined bases are those mutated and always lead to drug resistance; *p*^1^ was calculated use chi-square or fisher’s exact test ; *p*^2^ was calculated with Kruskal Wallis test.
